# Plasma lipid profiles discriminate bacterial from viral infection in febrile children

**DOI:** 10.1038/s41598-019-53721-1

**Published:** 2019-11-27

**Authors:** Xinzhu Wang, Ruud Nijman, Stephane Camuzeaux, Caroline Sands, Heather Jackson, Myrsini Kaforou, Marieke Emonts, Jethro A. Herberg, Ian Maconochie, Enitan D. Carrol, Stephane C. Paulus, Werner Zenz, Michiel Van der Flier, Ronald de Groot, Federico Martinon-Torres, Luregn J. Schlapbach, Andrew J. Pollard, Colin Fink, Taco T. Kuijpers, Suzanne Anderson, Matthew R. Lewis, Michael Levin, Myra McClure, EUCLIDS consortium, Stuart Gormley, Shea Hamilton, Bernardo Hourmat, Clive Hoggart, Vanessa Sancho-Shimizu, Victoria Wright, Amina Abdulla, Paul Agapow, Maeve Bartlett, Evangelos Bellos, Hariklia Eleftherohorinou, Rachel Galassini, David Inwald, Meg Mashbat, Stefanie Menikou, Sobia Mustafa, Simon Nadel, Rahmeen Rahman, Clare Thakker, Lachlan M. J. Coin, S. Bokhandi, Sue Power, Heather Barham, Dr N Pathan, Jenna Ridout, Deborah White, Sarah Thurston, S. Faust, S. Patel, Jenni McCorkell, P. Davies, Lindsey Crate, Helen Navarra, Stephanie Carter, R. Ramaiah, Rekha Patel, Catherine Tuffrey, Andrew Gribbin, Sharon McCready, Mark Peters, Katie Hardy, Fran Standing, Lauren O’Neill, Eugenia Abelake, Akash Deep, Eniola Nsirim, Louise Willis, Zoe Young, C. Royad, Sonia White, P. M. Fortune, Phil Hudnott, Fernando Álvez González, Ruth Barral-Arca, Miriam Cebey-López, María José Curras-Tuala, Natalia García, Luisa García Vicente, Alberto Gómez-Carballa, Jose Gómez Rial, Andrea Grela Beiroa, Antonio Justicia Grande, Pilar Leboráns Iglesias, Alba Elena Martínez Santos, Federico Martinón-Torres, Nazareth MartinónTorres, José María Martinón Sánchez, Beatriz Morillo Gutiérrez, Belén Mosquera Pérez, Pablo Obando Pacheco, Jacobo Pardo-Seco, Sara Pischedda, Irene RiveroCalle, Carmen Rodríguez-Tenreiro, Lorenzo Redondo-Collazo, Antonio Salas Ellacuriagal, Sonia Serén Fernández, María del Sol Porto Silva, Ana Vega, Lucía Vilanova Trillo, Antonio Salas, Susana Beatriz Reyes, María Cruz León León, Álvaro Navarro Mingorance, Xavier Gabaldó Barrios, Eider Oñate Vergara, Andrés Concha Torre, Ana Vivanco, Reyes Fernández, Francisco Giménez Sánchez, Miguel Sánchez Forte, Pablo Rojo, J. Ruiz Contreras, Alba Palacios, Cristina Epalza Ibarrondo, Elizabeth Fernández Cooke, Marisa Navarro, Cristina Álvarez Álvarez, María José Lozano, Eduardo Carreras, Sonia Brió Sanagustín, Olaf Neth, Ma del Carmen Martínez Padilla, Luis Manuel Prieto Tato, Sara Guillén, Laura Fernández Silveira, David Moreno, A. M. Tutu van Furth, N. P. Boeddha, G. J. A. Driessen, M. Emonts, J. A. Hazelzet, D. Pajkrt, E. A. M. Sanders, D. van de Beek, A. van der Ende, H. L. A. Philipsen, A. O. A. Adeel, M. A. Breukels, D. M. C. Brinkman, C. C. M. M. de Korte, E. de Vries, W. J. de Waal, R. Dekkers, A. Dings-Lammertink, R. A. Doedens, A. E. Donker, M. Dousma, T. E. Faber, G. P. J. M. Gerrits, J. A. M. Gerver, J. Heidema, J. Homan-van der Veen, M. A. M. Jacobs, N. J. G. Jansen, P. Kawczynski, K. Klucovska, M. C. J. Kneyber, Y. Koopman-Keemink, V. J. Langenhorst, J. Leusink, B. F. Loza, I. T. Merth, C. J. Miedema, C. Neeleman, J. G. Noordzij, C. C. Obihara, A. L. T. van Overbeek – van Gils, G. H. Poortman, S. T. Potgieter, J. Potjewijd, P. P. R. Rosias, T. Sprong, G. W. ten Tussher, B. J. Thio, G. A. Tramper-Stranders, M. van Deuren, H. van der Meer, A. J. M. van Kuppevelt, A. M. van Wermeskerken, W. A. Verwijs, T. F. W. Wolfs, Philipp Agyeman, Christoph Aebi, Christoph Berger, Philipp Agyeman, Christoph Aebi, Eric Giannoni, Martin Stocker, Klara M. Posfay-Barbe, Ulrich Heininger, Sara Bernhard-Stirnemann, Anita Niederer-Loher, Christian Kahlert, Paul Hasters, Christa Relly, Walter Baer, Christoph Berger, Hannah Frederick, Rebecca Jennings, Joanne Johnston, Rhian Kenwright, Elli Pinnock, Rachel Agbeko, Fatou Secka, Kalifa Bojang, Isatou Sarr, Ngange Kebbeh, Gibbi Sey, Momodou, Saidy khan, Fatoumata Cole, Gilleh Thomas, Martin Antonio, Daniela S. Klobassa, Alexander Binder, Nina A. Schweintzger, Manfred Sagmeister, Hinrich Baumgart, Markus Baumgartner, Uta Behrends, Ariane Biebl, Robert Birnbacher, Jan-Gerd Blanke, Carsten Boelke, Kai Breuling, Jürgen Brunner, Maria Buller, Peter Dahlem, Beate Dietrich, Ernst Eber, Johannes Elias, Josef Emhofer, Rosa Etschmaier, Sebastian Farr, Ylenia Girtler, Irina Grigorow, Konrad Heimann, Ulrike Ihm, Zdenek Jaros, Hermann Kalhoff, Wilhelm Kaulfersch, Christoph Kemen, Nina Klocker, Bernhard Köster, Benno Kohlmaier, Eleni Komini, Lydia Kramer, Antje Neubert, Daniel Ortner, Lydia Pescollderungg, Klaus Pfurtscheller, Karl Reiter, Goran Ristic, Siegfried Rödl, Andrea Sellner, Astrid Sonnleitner, Matthias Sperl, Wolfgang Stelzl, Holger Till, Andreas Trobisch, Anne Vierzig, Ulrich Vogel, Christina Weingarten, Stefanie Welke, Andreas Wimmer, Uwe Wintergerst, Daniel Wüller, Andrew Zaunschirm, Ieva Ziuraite, Veslava Žukovskaja

**Affiliations:** 1 Department of Infectious Disease, Imperial College London, London, W2 1PG United Kingdom; 2 National Phenome Centre and Imperial Clinical Phenotyping Centre, Department of Metabolism, Digestion and Reproduction, IRDB Building, Du Cane Road, Imperial College London, London, W12 0NN United Kingdom; 3 Great North Children’s Hospital, Paediatric Immunology, Infectious Diseases & Allergy, Newcastle upon Tyne Hospitals NHS Foundation Trust, Newcastle upon Tyne, NE1 4LP United Kingdom; 4 Translational and Clinical Research Institute, Newcastle University, Newcastle upon Tyne, NE2 4HH United Kingdom; 5 NIHR Newcastle Biomedical Research Centre based at Newcastle upon Tyne Hospitals NHS Trust and Newcastle University, Newcastle upon Tyne, NE4 5PL United Kingdom; 6 Department of Paediatric Emergency Medicine, St Mary’s Hospital, Imperial College NHS Healthcare Trust, London, W2 1NY United Kingdom; 7 Institute of Infection and Global Health, University of Liverpool, Liverpool, L69 7BE United Kingdom; 8 Department of Infectious Diseases, Alder Hey Children’s NHS Foundation Trust, Liverpool, L12 2AP United Kingdom; 9 Liverpool Health Partners, Liverpool, L3 5TF United Kingdom; 10 Department of General Paediatrics, Medical University of Graz, Graz, Auenbruggerplatz 34/2, 8036 Graz, Austria; 11 Pediatric Infectious Diseases and Immunology, Wilhelmina Children’s Hospital, University Medical Center Utrecht, Utrecht, 3508 AB The Netherlands; 12 Pediatric Infectious Diseases and Immunology, Amalia Children’s Hospital, and Section Pediatric Infectious Diseases, Laboratory of Medical Immunology, Department of Laboratory Medicine, Radboud Institute for Molecular Life Sciences, Radboud University Medical Center, Nijmegen, 6500 HB The Netherlands; 13 Genetic, Vaccines and Pediatric Infectious Diseases Research Group (GENVIP), Instituto de Investigación Sanitaria de Santiago and Universidad de Santiago de Compostela (USC), Galicia, Spain; 14 Translational Pediatrics and Infectious Diseases, Department of Pediatrics, Hospital Clínico Universitario de Santiago de Compostela, Galicia, 15706 Spain; 15 Paediatirc Criticial Care Research Group, Child Health Research Centre, The University of Queensland and Paediatric Intensive Care Research Group, Queensland Children’s Hospital, Brisbane, Australia; 16 Department of Paediatrics, University of Oxford and the NIHR Oxford Biomedical Research Centre, Oxford, OX3 9DU United Kingdom; 17 Micropathology Ltd, University of Warwick, Warwick, CV4 7EZ United Kingdom; 18 Division of Pediatric Hematology, Immunology and Infectious diseases, Emma Children’s Hospital Academic Medical Center, Amsterdam, 1105 AZ The Netherlands; 19 Medical Research Council Unit at the London School of Hygiene & Tropical Medicine, Banjul, The Gambia; 20 Poole Hospital NHS Foundation Trust, Poole, United Kingdom; 21 Cambridge University Hospitals NHS Trust, Cambridge, United Kingdom; 22 University Hospital Southampton, Southampton, United Kingdom; 23 Nottingham University Hospital NHS Trust, Nottingham, United Kingdom; 24 University Hospitals of Leicester NHS Trust, Leicester, United Kingdom; 25 Portsmouth Hospitals NHS Trust, Portsmouth, United Kingdom; 26 Great Ormond Street Hospital, London, United Kingdom; 27 King’s College Hospital NHS Foundation Trust, London, United Kingdom; 28 Oxford University Hospitals NHS Foundation Trust, Oxford, United Kingdom; 29 Kettering General Hospital NHS Foundation Trust, Kettering, United Kingdom; 30 Central Manchester NHS Trust, Manchester, United Kingdom; 31 Unidade de Xenética, Departamento de Anatomía Patolóxica e Ciencias Forenses, Instituto de Ciencias Forenses, Facultade de Medicina, Universidade de Santiago de Compostela, and GenPop Research Group, Instituto de Investigaciones Sanitarias (IDIS), Hospital Clínico Universitario de Santiago, Galicia, Spain; 32 Fundación Pública Galega de Medicina Xenómica, Servizo Galego de Saúde (SERGAS), Instituto de Investigaciones Sanitarias (IDIS), and Grupo de Medicina Xenómica, Centro de Investigación Biomédica en Red de Enfermedades Raras (CIBERER), Universidade de Santiago de Compostela (USC), Santiago de Compostela, Spain; 33 Hospital Clínico Universitario Virgen de la Arrixaca, Murcia, Spain; 34 Hospital de Donostia, San Sebastián, Spain; 35 Hospital Universitario Central de Asturias, Asturias, Spain; 36 Complejo Hospitalario Torrecárdenas, Almería, Spain; 37 Hospital Universitario 12 de Octubre, Madrid, Spain; 38 Hospital General Universitario Gregorio Marañón, Madrid, Spain; 39 Hospital de la Santa Creu i Sant Pau, Barcelona, Spain; 40 Hospital Universitario Virgen del Rocío, Sevilla, Spain; 41 Complejo Hospitalario de Jaén, Jaén, Spain; 42 Hospital Universitario de Getafe, Madrid, Spain; 43 Hospital Universitario y Politécnico de La Fe, Valencia, Spain; 44 Hospital Regional Universitario Carlos Haya, Málaga, Spain; 45 Vrije Universiteit University Medical Center, Amsterdam, The Netherlands; 46 Erasmus Medical Center – Sophia Children’s Hospital, Rotterdam, The Netherlands; 47 Institute of Cellular Medicine, Newcastle University, Newcastle upon Tyne, United Kingdom; 48 Paediatric Infectious Diseases and Immunology Department, Newcastle upon Tyne Hospitals Foundation Trust, Great North Children’s Hospital, Newcastle upon Tyne, United Kingdom; 49 University Medical Center Utrecht – Wilhelmina Children’s Hospital, Utrecht, The Netherlands; 50 Academic Medical Center, University of Amsterdam, Amsterdam, The Netherlands; 51 Kennemer Gasthuis, Haarlem, The Netherlands; 52 Elkerliek Hospital, Helmond, The Netherlands; 53 Alrijne Hospital, Leiderdorp, The Netherlands; 54 Beatrix Hospital, Gorinchem, The Netherlands; 55 Jeroen Bosch Hospital, ‘s-Hertogenbosch, The Netherlands; 56 Diakonessenhuis, Utrecht, The Netherlands; 57 Maasziekenhuis Pantein, Boxmeer, The Netherlands; 58 Gelre Hospitals, Zutphen, The Netherlands; 59 Martini Hospital, Groningen, The Netherlands; 60 Maxima Medical Center, Veldhoven, The Netherlands; 61 Gemini Hospital, Den Helder, The Netherlands; 62 Medical Center Leeuwarden, Leeuwarden, The Netherlands; 63 Canisius-Wilhelmina Hospital, Nijmegen, The Netherlands; 64 Rode Kruis Hospital, Beverwijk, The Netherlands; 65 St. Antonius Hospital, Nieuwegein, The Netherlands; 66 Deventer Hospital, Deventer, The Netherlands; 67 Slingeland Hospital, Doetinchem, The Netherlands; 68 Refaja Hospital, Stadskanaal, The Netherlands; 69 Bethesda Hospital, Hoogeveen, The Netherlands; 70 University Medical Center Groningen, Beatrix Children’s hospital, Groningen, The Netherlands; 71 Haga Hospital – Juliana Children’s Hospital, Den Haag, The Netherlands; 72 Isala Hospital, Zwolle, The Netherlands; 73 Bernhoven Hospital, Uden, The Netherlands; 74 VieCuri Medical Center, Venlo, The Netherlands; 75 Ziekenhuisgroep Twente, Almelo-Hengelo, The Netherlands; 76 Catharina Hospital, Eindhoven, The Netherlands; 77 Reinier de Graaf Gasthuis, Delft, The Netherlands; 78 ETZ Elisabeth, Tilburg, The Netherlands; 79 Scheper Hospital, Emmen, The Netherlands; 80 St. Jansdal Hospital, Hardewijk, The Netherlands; 81 Laurentius Hospital, Roermond, The Netherlands; 82 Isala Diaconessenhuis, Meppel, The Netherlands; 83 Zuyderland Medical Center, Sittard-Geleen, The Netherlands; 84 Westfriesgasthuis, Hoorn, The Netherlands; 85 Medisch Spectrum Twente, Enschede, The Netherlands; 86 St. Franciscus Gasthuis, Rotterdam, The Netherlands; 87 Streekziekenhuis Koningin Beatrix, Winterswijk, The Netherlands; 88 Flevo Hospital, Almere, The Netherlands; 89 Zuwe Hofpoort Hospital, Woerden, The Netherlands; 90 Department of Pediatrics, Inselspital, Bern University Hospital, University of Bern, Bern, Switzerland; 91 Service of Neonatology, Lausanne University Hospital, Lausanne, Switzerland; 92 Infectious Diseases Service, Lausanne University Hospital, Lausanne, Switzerland; 93 Department of Pediatrics, Children’s Hospital Lucerne, Lucerne, Switzerland; 94 Pediatric Infectious Diseases Unit, Children’s Hospital of Geneva, University Hospitals of Geneva, Geneva, Switzerland; 95 Infectious Diseases and Vaccinology, University of Basel Children’s Hospital, Basel, Switzerland; 96 Children’s Hospital Aarau, Aarau, Switzerland; 97 Division of Infectious Diseases and Hospital Epidemiology, Children’s Hospital of Eastern Switzerland St. Gallen, St. Gallen, Switzerland; 98 Department of Neonatology, University Hospital Zurich, Zurich, Switzerland; 99 Division of Infectious Diseases and Hospital Epidemiology, and Children’s Research Center, University Children’s Hospital Zurich, Zurich, Switzerland; 100 Children’s Hospital Chur, Chur, Switzerland; 101 Alder Hey Children’s Hospital, Clinical Research Business Unit, Eaton Road, Liverpool, L12 2AP United Kingdom; 102 Department of Pediatric and Adolescence Surgery, Division of General Pediatric Surgery, Medical University Graz, Graz, Austria; 103 Department of Pediatrics, General Hospital of Steyr, Steyr, Austria; 104 Department of Pediatrics/Department of Pediatric Surgery, Technische Universität München (TUM), Munich, Germany; 105 Department of Pediatrics, Kepler University Clinic, Medical Faculty of the Johannes Kepler University, Linz, Austria; 106 Department of Pediatrics and Adolesecent Medicine LKH Villach, Villach, Austria; 107 Department of Pediatrics and Adolescent Medicine and Neonatology, Hospital Ludmillenstift, Meppen, Germany; 108 Hospital for Children’s and Youth Medicine, Oberschwabenklinik, Ravensburg, Germany; 109 Department of Pediatrics, Medical University Innsbruck, Innsbruck, Austria; 110 Clinic for Paediatrics and Adolescents Medicine, Sana Hanse-Klinikum Wismar, Wismar, Germany; 111 Department of Pediatrics, Medical Center Coburg, Coburg, Germany; 112 University Medicine Rostock, Department of Pediatrics (UKJ), Rostock, Germany; 113 Department of Pulmonology, Medical University Graz, Graz, Austria; 114 Institute for Hygiene and Microbiology, University of Würzburg, Würzburg, Germany; 115 Clinical Institute of Medical and Chemical Laboratory Diagnostics, Medical University Graz, Graz, Austria; 116 Department of Pediatric Orthopedics and Adult Foot and Ankle Surgery, Orthopedic Hospital Speising, Vienna, Austria; 117 Department of Paediatrics, Regional Hospital Bolzano, Bolzano, Italy; 118 Department of Pediatrics and Adolescent Medicine, General Hospital Hochsteiermark/Leoben, Leoben, Austria; 119 Department of Neonatology and Paediatric Intensive Care, Children’s University Hospital, RWTH Aachen, Aachen, Germany; 120 Paediatric Intensive Care Unit, Department of Paediatric Surgery, Donauspital Vienna, Vienna, Austria; 121 Department of Pediatrics, General Public Hospital, Zwettl, Austria; 122 Pediatric Clinic Dortmund, Dortmund, Germany; 123 Department of Pediatrics and Adolescent Medicine, Klinikum Klagenfurt am Wörthersee, Klagenfurt, Austria; 124 Catholic Children’s Hospital Wilhelmstift, Department of Pediatrics, Hamburg, Germany; 125 Department of Pediatrics, Krankenhaus Dornbirn, Dornbirn, Austria; 126 Children’s Hospital Luedenscheid, Maerkische Kliniken, Luedenscheid, Germany; 127 Department of General Paediatrics, Medical University Graz, Graz, Austria; 128 Department of Paediatrics, Schwarzwald-Baar-Hospital, Villingen-Schwenningen, Germany; 129 Department of Paediatrics and Adolescents Medicine, University Hospital Erlangen, Erlangen, Germany; 130 Department of Pediatrics and Adolescent Medicine, Medical University of Salzburg, Salzburg, Austria; 131 Paediatric Intensive Care Unit, Medical University Graz, Graz, Austria; 132 Dr. von Hauner Children’s Hospital, Ludwig-Maximilians-Universitaet, Munich, Germany; 133 Mother and Child Health Care Institute of Serbia, Belgrade, Serbia; 134 Department of Pediatric and Adolescence Surgery, Division of Pediatric Orthopedics, Medical University Graz, Graz, Austria; 135 Department of Pediatrics, Academic Teaching Hospital, Landeskrankenhaus Feldkirch, Feldkirch, Austria; 136 University Children’s Hospital, University of Cologne, Cologne, Germany; 137 Department of Pediatrics and Adolescent Medicine Wilheminenspital, Vienna, Austria; 138 Department of Pediatric Surgery, Municipal Hospital Karlsruhe, Karlsruhe, Germany; 139 Hospital of the Sisters of Mercy Ried, Department of Pediatrics and Adolescent Medicine, Ried, Austria; 140 Hospital St. Josef, Braunau, Austria; 141 Christophorus Kliniken Coesfeld Clinic for Pediatrics, Coesfeld, Germany; 142 Department of Paediatrics, University Hospital Krems, Karl Landsteiner University of Health Sciences, Krems, Austria; 143 Children’s Hospital, Affiliate of Vilnius University Hospital Santariskiu Klinikos, Vilnius, Lithuania

**Keywords:** Metabolomics, Diagnostic markers

## Abstract

Fever is the most common reason that children present to Emergency Departments. Clinical signs and symptoms suggestive of bacterial infection are often non-specific, and there is no definitive test for the accurate diagnosis of infection. The ‘omics’ approaches to identifying biomarkers from the host-response to bacterial infection are promising. In this study, lipidomic analysis was carried out with plasma samples obtained from febrile children with confirmed bacterial infection (n = 20) and confirmed viral infection (n = 20). We show for the first time that bacterial and viral infection produces distinct profile in the host lipidome. Some species of glycerophosphoinositol, sphingomyelin, lysophosphatidylcholine and cholesterol sulfate were higher in the confirmed virus infected group, while some species of fatty acids, glycerophosphocholine, glycerophosphoserine, lactosylceramide and bilirubin were lower in the confirmed virus infected group when compared with confirmed bacterial infected group. A combination of three lipids achieved an area under the receiver operating characteristic (ROC) curve of 0.911 (95% CI 0.81 to 0.98). This pilot study demonstrates the potential of metabolic biomarkers to assist clinicians in distinguishing bacterial from viral infection in febrile children, to facilitate effective clinical management and to the limit inappropriate use of antibiotics.

## Introduction

Fever is the one of the most common reasons that children present to Emergency departments in hospitals, especially in children under 5 years of age, in England^1^ and in the US^2^. Serious bacterial infection accounts for 5–15% of the febrile children presenting^3–5^ and most cases originating from a viral aetiology are self-limiting. Currently bacterial infection is confirmed by positive microbiological culture of a sterile sample (blood, clean catch urine or cerebrospinal fluids (CSF)). However, this can take 24–48 hours and is compounded by having a high false-negative^4,6^ and false positive^7^ rates by contaminating pathogens. Molecular detection of specific pathogens is an option but results can be confounded by co-infections and samples need to be obtained from the site of infection which can be both invasive and impractical^8^. Because it is challenging for paediatricians to differentiate between bacterial and viral infection in acute illness, antibiotics are often prescribed as a precautionary measure, contributing to the rise of antimicrobial resistance.

It is clear that reliable biomarkers are urgently needed that distinguish bacterial from viral infection for the purpose of good clinical management and reducing antibiotic use. Host biomarkers, i.e. the physiological changes of the host in response to a specific pathogen, have untapped diagnostic potential and their discovery can be accelerated by the advances in ‘omics’ research, especially in the field of transcriptomics^9–12^ and proteomics^13–15^. Metabolomics has the added advantage that it is considered to most closely reflect the native phenotype and functional state of a biological system. One *In vivo* animal study revealed that distinct metabolic profiles can be derived from mice infected with different bacteria^16^ and several similar studies focusing on meningitis have shown that metabolic profiling of CSF can differentiate between meningitis and negative controls^17^, as well as between viral and bacterial meningitis^18^. Mason *et al*.^19^ demonstrated the possibility of diagnosis and prognosis of tuberculous meningitis with non-invasive urinary metabolic profiles. Metabolic changes in urine can be used to differentiate children with respiratory syncytial virus (RSV) from healthy control, as well as from those with bacterial causes of respiratory distress^20^.

Lipids are essential structural components of cell membranes and energy storage molecules. Thanks to the advances in lipidomics, a subset of metabolomics, lipids and lipid mediators have been increasingly recognised to play a crucial role in different metabolic pathways and cellular functions, particularly in immunity and inflammation^21,22^. However, the potential of lipidomics to distinguish bacterial from viral infection in febrile children has never been explored.

In this study, we undertook a lipidomic analysis of plasma taken from febrile children with confirmed bacterial infection (n = 20) and confirmed viral infection (n = 20) as a proof of concept study. We show that bacterial and viral infection produces distinct profiles in the plasma lipids of febrile children that might be exploited diagnostically.

## Methods

### Study population and sampling

The European Union Childhood Life-Threatening Infectious Disease Study (EUCLIDS)^23^ prospectively recruited patients, aged from 1 month to 18 years, with sepsis or severe focal infection from 98 participating hospitals in the UK, Austria, Germany, Lithuania, Spain and the Netherlands between 2012 and 2015. Plasma and other biosamples were collected to investigate the underlying genetics, proteomics and metabolomics of children with severe infectious disease phenotype.

Infections in Children in the Emergency Department (ICED) study aimed to define clinical features that would predict bacterial illness in children and patterns of proteomics, genomics and metabolomics associated with infections. This study included children aged 0–16 years at Imperial College NHS Healthcare Trust, St Mary’s Hospital, between June 2014 and March 2015^24^.

The population consisted of children (≤17 years old) presenting with fever ≥38 °C, with diverse clinical symptoms and a spectrum of pathogens. Both studies were approved by the local institutional review boards (ICED REC No 14/LO/0266 approved by NRES Committee London – Camden & Islington; EUCLIDS REC No 11/LO/1982 approved by NRES Committee London – Fulham). Written informed consent was obtained from parents and assent from children, where appropriate. All methods were performed in accordance with the relevant guidelines and regulations. For the EUCLIDS study, a common clinical protocol agreed by EUCLIDS Clinical Network and approved by the Ethics Committee was implemented at all hospitals.

Patients were divided into those with confirmed bacterial (n = 20) and confirmed viral (n = 20) infection groups. The bacterial group consisted exclusively of patients with confirmed sterile site culture-positive bacterial infections, and the viral infection group consisted of only patients with culture, molecular or immunofluorescent-confirmed viral infection and having no co-existing bacterial infection.

Blood samples were collected in tubes spray-coated with EDTA at, or as close as possible to, the time of presentation to hospital and plasma obtained by centrifugation of blood samples for 10 mins at 1,300 g at 4 °C. Plasma was stored at −80 °C before being shipped on dry ice to Imperial College London for lipidomic analysis.

### Lipidomic analysis

Lipidomic analysis was carried out as previously described^25^. Briefly, 50 µl of water were added to 50 µl of plasma, vortexed and shaken for 5 min at 1,400 rpm at 4 °C. Four hundred µl of isopropanol containing internal standards (9 in negative mode, 11 for positive mode covering 10 lipid sub-classes) were added for lipid extraction. Samples were shaken at 1,400 rpm for 2 hours at 4 °C then centrifuged at 3,800 g for 10 min. Two aliquots of 100 µl of the supernatant fluid were transferred to a 96-well plate for ultra-performance liquid chromatography (UPLC) –mass spectrometry (MS) lipidomics analysis in positive and negative mode.

Liquid chromatography separation was carried out using an Acquity UPLC system (Waters Corporation, USA) with an injection volume of 1 µl and 2 µl for Positive and Negative ESI, respectively. An Acquity UPLC BEH column (C8, 2.1 × 100 mm, 1.7 µm; Waters Corporation, USA) was used for the purpose. Mobile phase A consisted of water/isopropanol/acetonitrile (2:1:1; v:v:v) with the addition of 5 mM ammonium acetate, 0.05% acetic acid and 20 µM phosphoric acid. Mobile phase B consisted of isopropanol: acetonitrile (1:1; v:v) with the addition of 5 mM ammonium acetate and 0.05% acetic acid. Flow rate was 0.6 ml/min with a total run time of 15 min and the gradient set as starting condition of 1% mobile phase B for 0.1 min, followed by an increase to 30% mobile phase B from 0.1 to 2 min, and to 90% mobile phase B from 2 min to 11.5 min. The gradient was held at 99.99% mobile phase B between 12 and 12.55 min before returning to the initial condition for re-equilibrium.

MS detection was achieved using a Xevo G2-S QTof mass spectrometer (Waters MS Technologies, UK) and data acquired in both positive and negative modes. The MS setting was configured as follows: capillary voltage 2.0 kV for Positive mode, 1.5 kV for Negative mode, sample cone voltage 25 V, source offset 80, source temperature 120 °C, desolvation temperature 600 °C, desolvation gas flow 1000 L/h, and cone gas flow 150 L/h. Data were collected in centroid mode with a scan range of 50–2000 m/z and a scan time of 0.1 s. LockSpray mass correction was applied for mass accuracy using a 600 pg/ µL leucine enkephaline (m/z 556.2771 in ESI+, m/z 554.2615 in ESI−) solution in water/acetonitrile solution (1:1; v/v) at a flow rate of 15 µl/min.

### Spectral and statistical analysis

A Study Quality Control sample (SQC) was prepared by pooling 25 µl of all samples. The SQC was diluted to seven different concentrations, extracted at the same ratio 1:4 with isopropanol and replicates acquired at each concentration at the beginning and end of the run. A Long-Term Reference sample (LTR, made up of pooled plasma samples from external sources) and the SQC were diluted with water (1:1; v:v) and 400 µL of isopropanol containing internal standards (the same preparation as for the study samples) and injected once every 10 study samples, with 5 samples between a LTR and a SQC. Deconvolution of the spectra was carried out using the XCMS package. Extracted metabolic features were subsequently filtered and only those present with a relative coefficient of variation less than 15% across all SQC samples were retained. Additionally, metabolic features that did not correlate with a coefficient greater than 0.9 in a serial dilution series of SQC samples were removed.

Multivariate data analysis was carried out using SIMCA-P 14.1 (Umetrics AB, Sweden). The dataset was pareto-scaled prior to principal component analysis (PCA) and orthogonal partial least squares discriminate analysis (OPLS-DA). While PCA is an unsupervised technique useful for observing inherent clustering and identifying potential outliers in the dataset, OPLS-DA is a supervised method in which data is modelled against a specific descriptor of interest (in this case viral vs. bacterial infection classes). As for all supervised methods, model validity and robustness must be assessed before results can be interpreted. For OPLS-DA, model quality was assessed by internal cross-validation (Q^2^Y-hat value) and permutation testing in which the true Q^2^Y-hat value is compared to 999 models with random permutations of class membership. For valid and robust models (positive Q^2^Y-hat and permutation p-value < 0.05), metabolic features responsible for class separation were identified by examining the corresponding S-plot (a scatter plot of model loadings and correlation to class) with a cut-off of 0.05.

### Metabolite annotation

Short-listed metabolic features were subjected to tandem mass spectrometry in order to obtain fragmentation patterns. Patterns were compared against metabolome databases (Lipidmaps, HMDB, Metlin). Isotopic distribution matching was also checked. In addition, when possible the fragmented patterns were matched against available authentic standards run under the same analytical setting for retention time and MS/MS patterns. Annotation level, according to the Metabolomics Standards Initiative, are summarised in Table 1 ^26^.

**Table 1:** Metabolic features changed in bacterial and viral group.

	m/z	Retention time	Annotation	Annotation level	Ion type	Neutral formula
Lipids/metabolites increased in bacterial infected group	279.231	2.52	FA(18:2)	2	[M − H]-	C18H32O2
	255.232	2.82	FA(16:0)	2	[M − H]-	C16H32O2
	281.247	2.96	FA(18:1)	2	[M − H]-	C18H34O2
	788.545	6.22	PS(18:0/18:1)	2	[M − H]-	C39H74NO8P
	253.216	2.35	FA(16:1)	2	[M − H]-	C16H30O2
	742.54	6.06	PC(16:0/18:2)	2	[M − CH3]-	C42H80NO8P
	716.524	6.75	PE(16:0/18:1)	2	[M − H]-	C39H76NO8P
	583.256	1.18	Bilirubin	2	[M − H]-	C33H36N4O6
	810.53	5.76	PS(18:0/20:4)	2	[M − H]-	C44H78NO10P
	846.624	7.23	PC(18:0/18:1)	2	[M + PO4H2]-	C44H86NO8P
	1068.7	7.80	LacCer(d18:1/24:1)	2	[M + PO4H2]-	C54H101NO13
	770.571	6.78	PC(18:0/18:2)	2	[M − CH3]-	C44H84NO8P
	744.556	6.60	PC(16:0/18:1)	2	[M − CH3]-	C42H82NO8P
	958.589	5.78	LacCer(d18:1/16:0)	2	[M + PO4H2]-	C46H87NO13
	718.54	6.41	PC(16:0/16:0)	2	[M − CH3]-	C40H80NO8P
	742.54	6.90	PE(18:0/18:2)	2	[M − H]-	C41H78NO8P
Lipids/metabolites increased in viral infected group	465.303	2.55	Cholesterol sulfate	2	[M − H]-	C27H46O4S
	465.303	2.61	Cholesterol sulfate	2	[M − H]-	C27H46O4S
	909.551	5.56	PI(18:0/22:6)	2	[M − H]-	C49H83O13P
	861.55	5.75	PI(18:0/18:2)	2	[M − H]-	C45H83O13P
	797.655	7.78	SM(d18:1/24:1)	2	[M − CH3]-	C47H93N2O6P
	339.231	2.66	UNKNOWN1	4		
	772.529	6.49	PE1	3	[M − H]-	C45H76NO7P
	897.648	8.12	SM(d18:1/23:0)	2	[M + PO4H2]-	C46H93N2O6P
	239.157	0.87	UNKNOWN2	4		
	886.609	6.31	SHexCer(d42:3)	2	[M − H]-	C48H89NO11S
	554.346	1.86	LPC(16:0/0:0)	2	[M + CH3COO]-	C24H50NO7P
	799.671	8.41	SM(d18:1/24:0)	2	[M − CH3]-	C47H95N2O6P
	750.545	7.24	PE2	3	[M − H]-	C41H78NO7P

FA: fatty acid; PE: glycerophosphotidy-lethanolamine; PC: glycerophosphocholine; PS: glycerophosphoserine; LacCer: lactosylceramide; PI: glycerophosphoinositol; SM: sphingomyelin; LPC: Lysophosphatidylcholine; SHexCer: Sulfatides.

### Single feature ROC curve analysis

Analysis was performed with the web server, MetaboAnalyst 4.0. Sensitivities and specificities of lipids and predicted probabilities for the correct classification were presented as Receiver Operating Characteristic (ROC) curves. The Area Under the Curve (AUC) represents the discriminatory power of the lipids, with the value closest to 1 indicating the better classification.

### Feature selection

An ‘in-house’ variable selection method, forward selection-partial least squares (FS-PLS; https://github.com/lachlancoin/fspls.git), was used to identify a small diagnostic signature for distinguishing bacterial and viral infections. FS-PLS identifies a small signature made up non-correlated features. The first iteration of FS-PLS considers the levels of all features (N) and initially fits N univariate regression models. The regression coefficient for each model is estimated using the Maximum Likelihood Estimation (MLE) function, and the goodness of fit is assessed by a t-test. The variable with the highest MLE and smallest p-value is selected first (SV1). Before selecting which of the N-1 remaining variables to use next, the algorithm projects the variation explained by SV1 using Singular Value Decomposition (SVD). The algorithm iteratively fits up to N-1 models, at each step projecting the variation corresponding to the already selected variables, and selecting new variables based on the residual variation. Projecting out the variation of selected features ensures that the final features in the signature are not correlated with each other. The FS-PLSprocess terminates when the MLE p-value exceeds a pre-defined threshold (p_thresh_). The final model includes regression coefficients for all selected variables. First, FS-PLS was applied to the abundance values for the short-listed metabolic features identified through OPLS-DA analysis.

An individual’s age and sex can impact upon their metabolome greatly. Limma^27^ was used for differential abundance analysis to identify metabolites that are associated with age or sex. Features were considered to be associated with age or sex if they achieved an FDR p-value lower than 0.05. FS-PLS was re-applied to the dataset after having removed these features and the resulting signature was compared to the signatures from the full dataset.

The disease risk score (DRS) was calculated for the 3 metabolite signature. The DRS translates the abundance values of the features in the signature into a single value, indicating the disease group of the sample^11^.

The sensitivity and specificity of the lipid signature were presented as a receiver operating characteristic (ROC) curve with the 95% confidence regions calculated through bootstrap analysis with 500 iterations.

## Results

### Patient characteristics

The baseline characteristics were divided into those with definitive bacterial and definitive viral infection, summarised in Table 2. When selecting patient samples, patient characteristics were matched as much as possible to ensure no particular factor would confound the model. There was no significant difference in ages between the two groups (p = 0.97). Both groups had similar sex split. Seven from definitive bacterial infection group and 6 from the definitive viral infection group were admitted to the Paediatric Intensive Care Unit (PICU). A range of pathogens was present in each group.

**Table 2:** Demographic and clinical patient characteristic.

Patients with confirmed	Bacterial infection (N = 20)	Viral infection (N = 20)	P value
Age, median (range), month	9 (1–102)	8 (1–93)	p = 0.48
Male, No. (%)	11 (55)	10 (50)	—
White race, No./total (%)	14/19 (74)	11/20 (55)	—
Time from symptoms to blood sampling, median (range), day	2 (0–9)	3 (0–15)	p = 0.16
Intensive care, No. (%)	7 (35)	6 (30)	—
Fatalities, No.	1	0	—
Pathogen* (#cases)	Coliform (1) *B. pertussis* (2) *E. coli* (2) *S. Pneumoniae* (3) *S. aureus* (1) *E. cloacae* (1) *N. Meningitidis (8)* *K. Kingae* (1) *Klebsiella oxytoca* (1) *Group A streptococcus* (1)**	Enterovirus (3) Influenza A (2) Parechovirus (1) Respiratory syncytial virus (5) Rhinovirus (3) Adenovirus (4) Human Metapneumovirus (1) Parainfluenza virus (1) Human herpesvirus 6 (1) Herpes simplex virus (1) Rotavirus (1)	
Source of the samples	St. Mary’s Hospital (2) Alder Hey Children’s NHS Foundation (3) Poole Hospital NHS Foundation Trust (2) Nottingham University Hospitals (2) Medical University of Graz (1) General Hospital of Leoben (1) Hospital Clinico Univeritario de Santiago (5) Hospital Universitario 12 de Octubre (2) Complejo Hospitalario de Jaen (1) Erasms MC (1)	St Mary’s Hopsital (11) Newcastle Upon Tyne Hospitals NHS (1) Cambridge University Hospitals NHS Foundation Trust (2) Great Ormond Street Hospital (1) Nottingham University Hospitals (2) Hospital Clinico Univeritario de Santiago (2) Erasmus MC (1)	

*Some patients are co-infected with more than one pathogen.

**The patient with Group A *streptococcus* was excluded from the subsequent data analysis as being an outlier.

### Plasma lipidome can differentiate bacterial from viral infection

**Figure 1:**
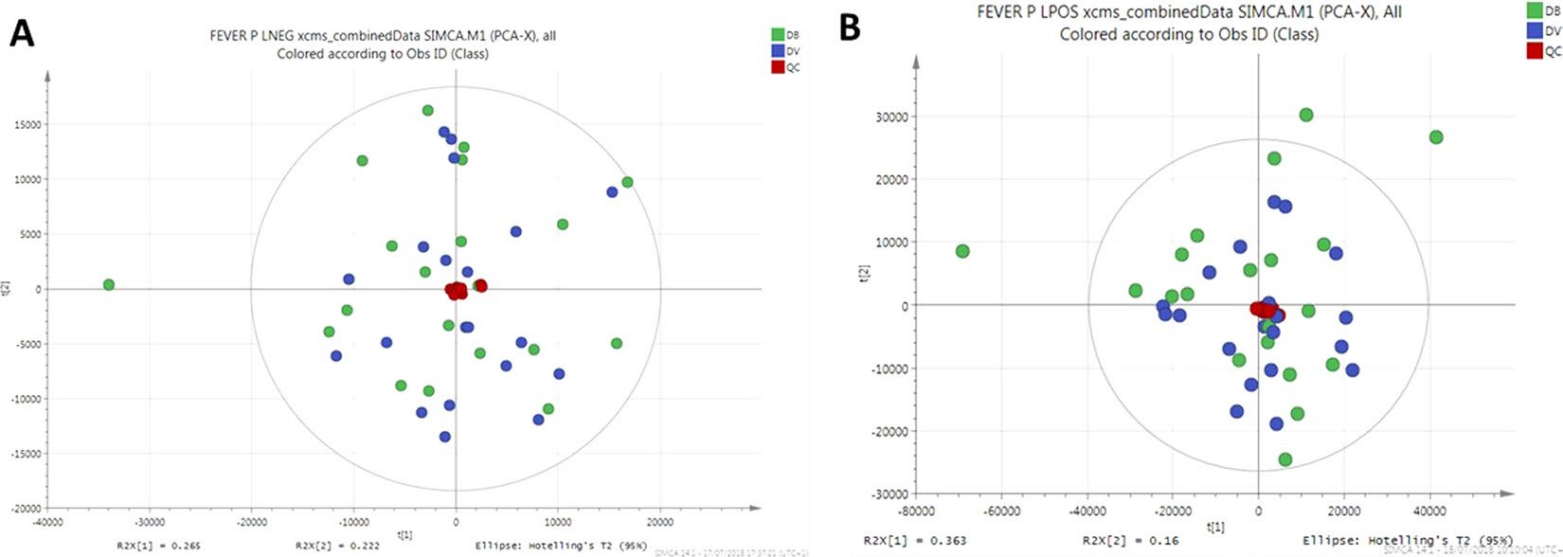
Principal components analysis (PCA) of lipidomics dataset. (**A**) Scatter plot of PCA model from data acquired in negative polarity mode. (**B**) Scatter plot of PCA model from data acquired in positive polarity mode. Quality control samples are shown in red, bacterial infected samples are shown in blue and viral infected samples shown in green.

PCA was conducted first to evaluate the data, visualise dominant patterns, and identify outliers within populations (Fig. 1). The same outlier sample was present in both negative (Fig. 1A) and positive (Fig. 1B) polarity datasets and as such, was removed from subsequent analysis. SQC samples were tightly grouped together in the PCA scatter plot, indicating minimum analytical variability throughout the run.

**Figure 2:**
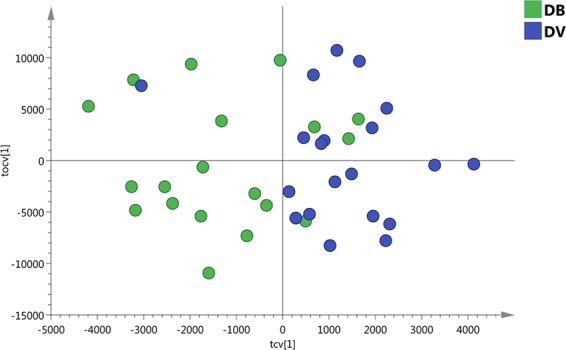
The scatter plot of the cross-validated score vectors showing the clustering of definitive bacterial infected samples (green dots) from definitive viral infected samples (blue dots).

OPLS-DA, a supervised PCA method, was carried out on both positive and negative polarity datasets. In the positive polarity mode no model was successfully built to distinguish between viral and bacterial infection groups (data not shown). However, in the negative polarity dataset, an OPLS-DA model separated bacterial infected samples from viral infected samples (with 3891 features). The robustness of the model was characterised by R2X (cum) = 0.565, R2Y-hat (cum) = 0.843 and Q2Y-hat (cum) = 0.412 and permutation p-value = 0.01 (999 tests). Cross-validated scores plot using the whole lipidome dataset indicated bacterial infected samples were more prone to miss-classification than viral infected samples (Fig. 2).

### Lipid changes were not the same in the bacterial and viral infected groups

**Figure 3:**
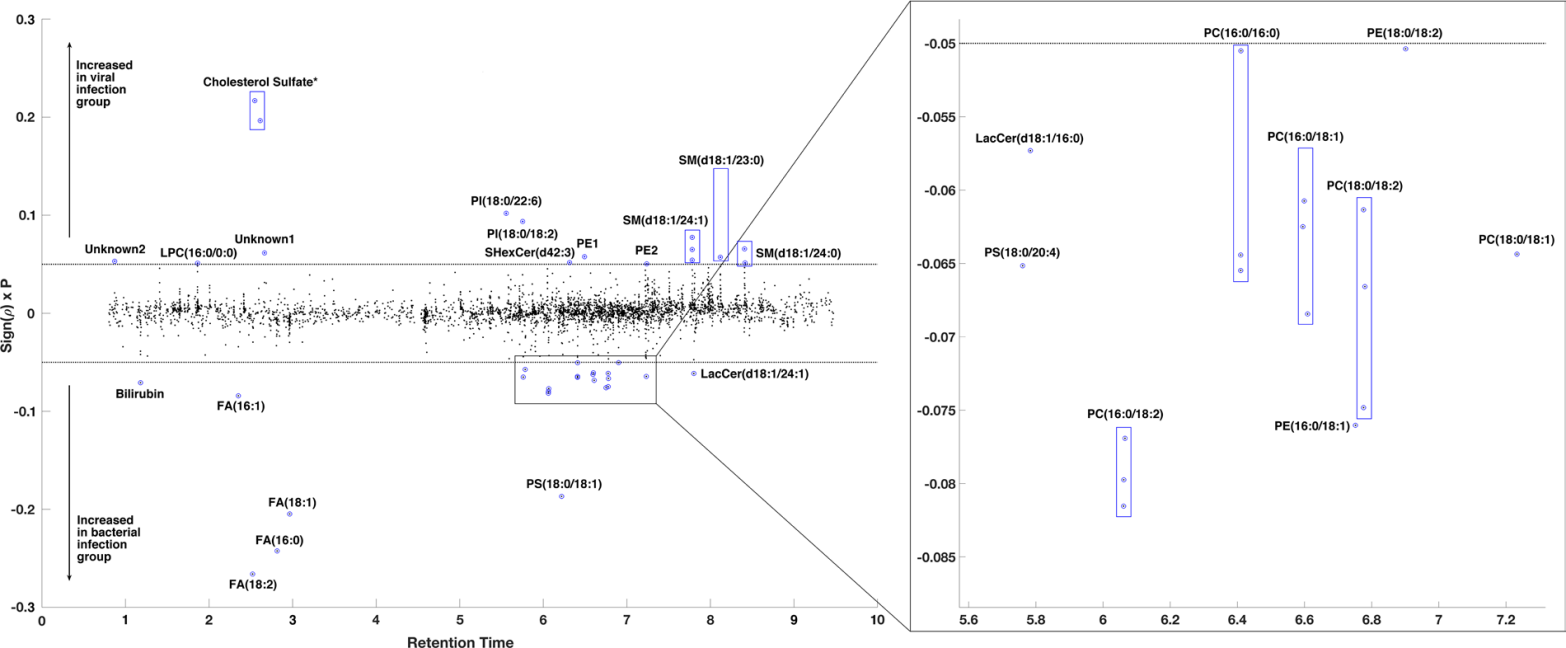
Manhattan-style plot of the 3891 lipid features detected by lipid-positive mode UPLC-MS with 40 features showing a significant association with infection type (as determined by model S-plot) highlighted and annotated. Y axis Sign(*p*) x P is the loadings of the OPLS-DA (i.e. modelled covariance p[1]). *Cholesterol sulfate – isomers due to different position of the sulfate.

Metabolic features contributing to the separation of the model are plotted in Fig. 3 and summarised in Table 1. Some species of glycerophosphoinositol, monoacylglycerophosphocholine, sphingomyelin and sulfatide were higher in the viral group when compared to the bacterial group, while some species of fatty acids, glycerophosphocholine, glycerophosphoserine and lactosylceramide were higher in bacterial infection when compared with viral infection. Bilirubin and cholesterol sulfate, although not lipids, were detected by lipidomic analysis, and these were higher in the bacterial and viral groups when compared to the other group, respectively.

### Evaluation of diagnostic potential of metabolic biomarkers

**Figure 4:**
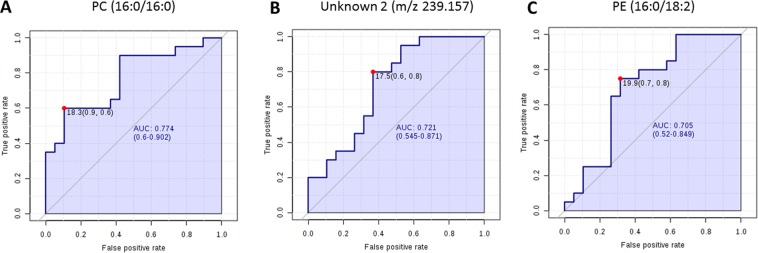
Receiver operator characteristic (ROC) analysis based on single lipids. ROC curve analysis of top 3 lipids PC (16:0/16:0) (**A**), unknown feature (m/z 239.157) (**B**) and PE (16:0/18:2) (**C**) which gave with highest Area Under the Curve (AUC) values.

ROC curve analysis was performed to evaluate the diagnostic potential of these lipids in distinguishing bacterial from viral infection. Out of all discriminatory lipids, PC (16:0/16:0), unknown feature m/z 239.157 and PE (16:0/18:2) generated the highest AUCs of 0.774 (CI, 0.6–0.902), 0.721 (CI, 0.545–0.871) and 0.705 (CI, 0.52–0.849), respectively (Fig. 4).

FS-PLS was initially carried out on the abundance values for all 28 shortlisted features. A signature was identified made up the following 3 lipids: SHexCer(d42:3); PC (16:0/16:0); and LacCer(d18:1/24:1). The impacts of age and sex on the feature selection process were explored. With a false discovery rate (FDR) of 0.05, 5 out of the 28 features were identified as being significantly differentially abundant between samples above or below the median age. None of the 28 features were identified as being significantly differentially abundant between males and females. The 5 features that were associated with age were removed and FS-PLS was re-ran on the filtered dataset. The same 3-metabolite signature (SHexCer(d42:3), PC(16:0/16:0), LacCer(d18:1/24:1)) was identified, showing that the signature is robust to age effects.

**Figure 5:**
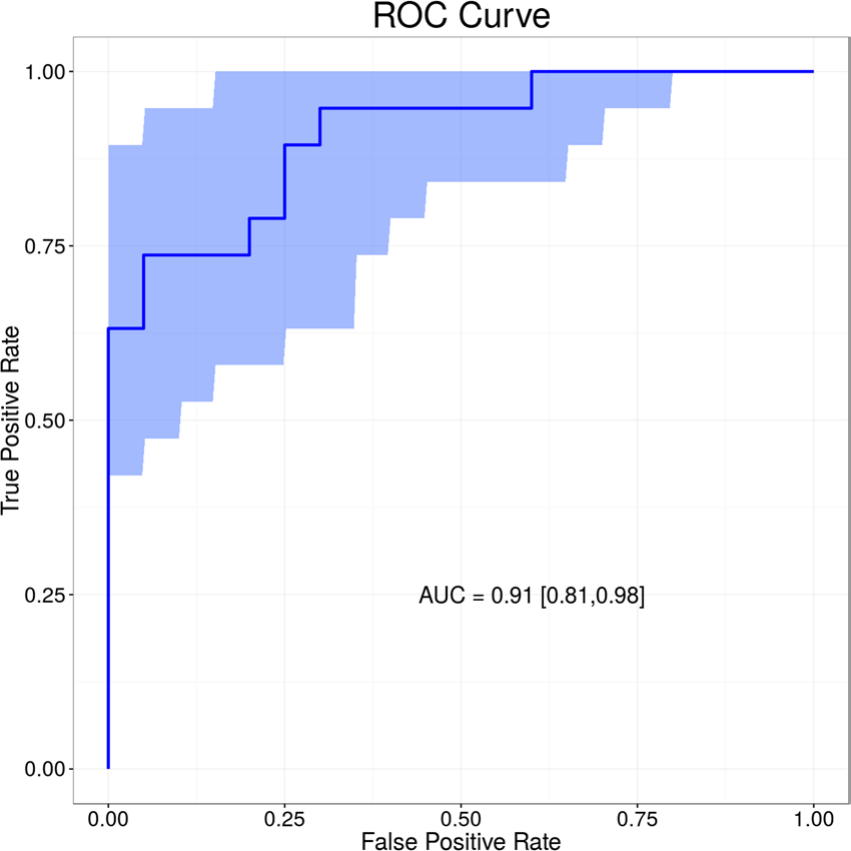
Receiver operator characteristic (ROC) analysis based on 3-lipid signature. A combination of SHexCer(d42:3), PC (16:0/16:0) and LacCer(d18:1/24:1) achieved AUC of 0.911 (CI 95% 0.81–0.98).

**Figure 6:**
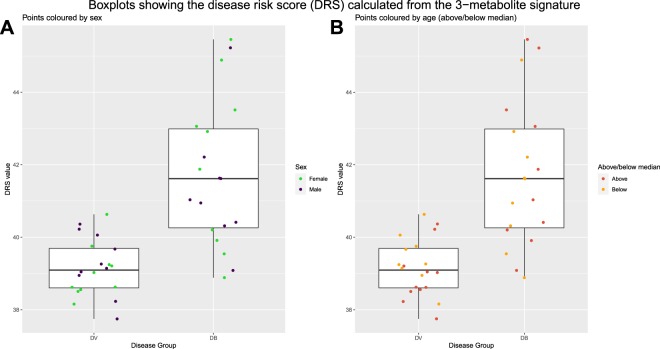
Boxplots comparing the Disease Risk Score (DRS) for definitive bacterial and definitive viral samples. The DRS was calculated using abundance values from the 3-metabolite signature identified by FS-PLS. Plot A shows points coloured according to the sex of the sample and plot B shows points coloured according to whether the sample was above or below the median age (9 months).

This signature achieved an improved ROC curve with AUC of 0.911 (95% confidence interval: 0.81–0.98) when compared with those generated from individual lipids. The ROC curve and confidence intervals calculated through bootstrapping are shown in Fig. 5. Figure 6 shows the disease risk scores for definitive bacterial and definitive viral samples with points overlaid to indicate the sex or age (above or below median) of the sample.

## Discussion

We have shown that differences in the host lipidome are induced by bacterial and viral infections. While differences in host responses between viral and bacterial infections have been previously reported, for example as differential expression of proteins, RNAs and level of metabolites^9–14,20^, there have been no claims in relation to the lipidome changes in carefully-phenotyped samples. Although age is known to affect metabolism^28^, it is important to note the metabolic changes associated with infection described herein, were consistent among samples from patients whose age ranged from 1 month to 9 years old.

Some species of glycerophosphoinositol, sphingomyelin, lysophosphatidylcholine and cholesterol sulfate were higher in the confirmed virus-infected group when compared with bacterial infected group, while some species of fatty acids, glycerophosphocholine, glycerophosphoserine, lactosylceramide and bilirubin were higher in cases with confirmed bacterial infection when compared with viral infection.

The important effects of infection on fatty acid metabolism have been highlighted by Munger *et al*. who demonstrated human cytomegalovirus (HCMV) up-regulated fatty acid biosynthesis in infected host cells. Pharmacologically inhibition of fatty acid biosynthesis suppressed viral replication for both HCMV and influenza A virus^29^. The importance of fatty acid biosynthesis may reflect its essential role in viral envelopment during viral replication. Rhinovirus induced metabolic reprogramming in host cell by increasing glucose uptake and indicated a shift towards lipogenesis and/or fatty acid uptake^30^. In our study, fatty acids linoleic acid (FA 18:2), palmitic acid (FA 16:0), oleic acid (FA 18:1) and palmitoleic acid (FA 16:1) were lower in viral infection when compared to bacterial infection, and may reflect enhanced lipogenesis and fatty acid uptake in the host cell during viral replication.

The increase in cholesterol sulfate observed may reflect changes in cellular lipid biosynthesis and T cell signalling during viral infection. Cholesterol sulfate is believed to play a key role as a membrane stabiliser^31^ and can also act to modulate cellular lipid biosynthesis^32^ and T cell receptor signal transduction^33^. Gong *et al*. demonstrated that cholesterol sulfate was elevated in the serum of piglets infected with swine fever virus^34^. Taken together, these observations indicate that this compound could be a marker of viral infection.

Higher level of sphingomyelin SM(d18:1/24:1), SM(d18:1/23:0) and SM(d18:1/24:0), and lysophosphocholine LPC (16:0) upon viral infection may also be linked to viral replication in infected cells. Accumulation of cone-shaped lipids, such as LPC in one leaflet of the membrane bilayer induces membrane curvature required for virus budding^35^. It is known that viral replication, for example in the case of dengue virus, induces dramatic changes in infected cells, including sphingomyelin, to alter the curvature and permeability of membranes^36^. Furthermore, the altered levels of sphingomyelin can be partially explained by elevated cytokine levels during bacterial infection, such as TNF-α^37^, which can activate sphingomyelinase, hydrolysing sphingomyelin to ceramide^38^. Hence, sphingomyelin may be a class of lipids that plays a role in both viral and bacterial infection.

Lactosylceramide LacCer(d18:1/24:1) and LacCer (d18:1/16:0) were higher in bacterial infection in comparison to viral infection. Lactosylceramide, found in microdomains on the plasma membrane of cells, is a glycosphingolipid consisting of a hydrophobic ceramide lipid and a hydrophilic sugar moiety. Lactosylceramide plays an important role in bacterial infection by serving as a pattern recognition receptors (PRRs) to detect pathogen-associated molecular patterns (PAMPs). Lactosylceramide composed of long chain fatty acid chain C24, such as LacCer(d:18:1/24:1) increased in our study, is essential for formation of LacCer-Lyn complexes on neutrophils, which function as signal transduction platforms for αMβ2 integrin-mediated phagocytosis^39^.

Other lipids that were changed in our study, such as sulfatides and glycerophosphocholines, may also play an important role in bacterial infection. Sulfatides are multifunctional molecules involved in various biological process, including immune system regulation and during infection^40^. Sulfatides can act as glycolipid receptors that attach bacteria, such as *Escherichia coli*^41^, *Mycoplasma hyopneumoniae*^42^ and *Pseudomonas aeruginosa*^43^ to the mucosal surfaces. Five glycerophosphocholine species including PC(16:0/18:2), PC(18:0/18:1), PC(18:0/18:2), PC(16:0/16:0) and PC(16:0/18:1) werehigher in bacterial infected samples when compared with viral infected samples. Glycerophosphocholine was elevated in a lipidomics study looking at plasma from tuberculosis patients^44^, however, the exact role of glycerophosphocholine remains elusive. Bilirubin is detected as a consequence of breadth of lipidome coverage, and its role in infection is unclear. The lipid species identified in this study present an opportunity for further mechanistic study to understand the host responses in bacterial or viral infection.

A combination of three lipids achieved a strong area under the receiver operating characteristic (ROC) curve of 0.911 (95% CI 0.81 to 0.98). Similar approaches have been taken using routine laboratory parameters and more recently gene expression where 2-gene transcripts achieved an ROC AUC of 0.95 (95% CI 0.94-1)^11^. The relevance of our data is that they provide the potential for a rapid diagnostic test with which clinicians could distinguish bacterial from viral infection in febrile children.

The study has limitations. Firstly, we were unable to annotate 4 of the 29 discriminatory features, of which two were assigned with only a broad lipid class by identifying the head group (PE). The unknown feature with m/z of 239.157 achieved the second highest AUC for ROC curve analysis on an individual basis. The unknown identity prevents this feature from being a potential marker and hinders biological understanding. This feature, however, was not included in the final 3-lipid panel that gave the highest AUC. Secondly, the sample size in this pilot study is small. Validation studies using quantitative assay are now required to confirm the findings. In addition, in larger validation studies, we will look into the signature of specific pathogens, and potentially co-infection by multiple pathogens.

This is the first lipidomics study carried out on plasma taken from febrile children for the purpose of distinguishing bacterial from viral infection. It demonstrates the potential of this approach to facilitate effective clinical management by rapidly diagnosing bacterial infection in paediatrics.

## Data Availability

The datasets generated and/or analysed during the current study are available from corresponding author on a reasonable request.
